# Microneedle-Mediated Transdermal Delivery of Drug-Carrying Nanoparticles

**DOI:** 10.3389/fbioe.2022.840395

**Published:** 2022-02-11

**Authors:** Xue Jiang, Huanhuan Zhao, Wei Li

**Affiliations:** Key Laboratory of Combinatorial Biosynthesis and Drug Discovery (Ministry of Education), School of Pharmaceutical Sciences, Wuhan University, Wuhan, China

**Keywords:** microneedle, transdermal delivery, nanoparticles, controlled release, drug delivery

## Abstract

Drug-carrying nanoparticles have obtained great attention for disease treatments due to the fact that they can improve drug solubility, provide drug protection and prolong release duration, thus enhancing drug bioavailability and increasing therapeutic efficacy. Although nanoparticles containing drugs can be administered *via* different routes such as oral, intravenous and ocular, transdermal delivery of nanoparticles mediated by microneedles has attracted considerable interest due to the capability of circumventing enzymatic degradation caused by gastrointestinal track, and increasing patient compliance by reducing pain associated with hypodermic injection. In this review, we first introduce four types of nanoparticles that were used for drug delivery, and then summarize strategies that have been employed to facilitate delivery of drug-loaded nanoparticles *via* microneedles. Finally, we give a conclusion and provide our perspectives on the potential clinical translation of microneedle-facilitated nanoparticles delivery.

## Introduction

**FIGURE 1:**
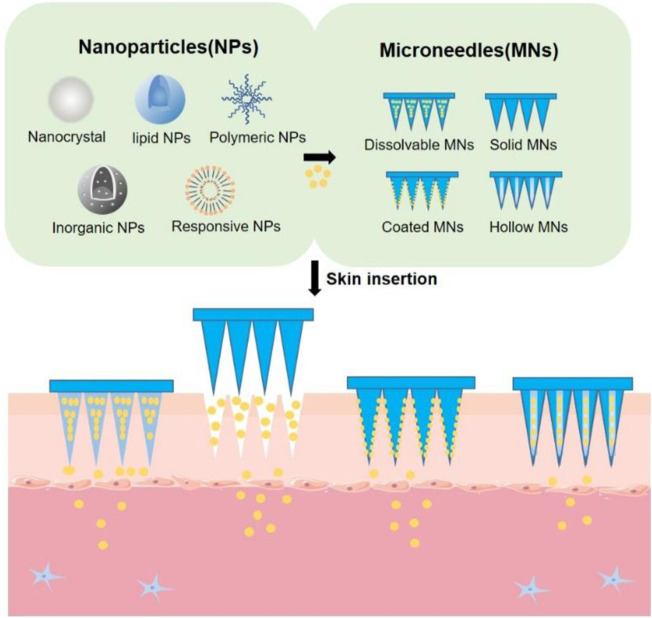
The schematic illustration of MNs-mediated transdermal delivery of nanoparticles.

Due to the unique advantages, such as protection from enzyme degradation, prolonged half-life of drugs, desired targetability, ability to achieve sustained release, nanoparticles have been extensively used for delivering a wide variety of drugs that are applied for multiple disease treatments, such as diabetes, wound healing and cancers (Baetke et al., 2015). Although drug-loaded nanoparticles can be administered *via* different routes for therapies such as oral, intravenous and ocular administration, transdermal delivery of nanoparticles mediated by microneedles (MNs) has drawn considerable attention due to the capability of circumventing enzymatic degradation caused by gastrointestinal track, and increasing patient compliance by reducing pain associated with hypodermic injection (Table 1). MNs are array of micro-scale needles that can penetrate the outmost skin layer, termed stratum corneum, and enter the skin to achieve transdermal drug delivery in a minimally invasive manner (Prausnitz, 2017). Using MNs, many kinds of drugs have been successfully and efficiently delivered into the skin, such as levonorgestrel (Li et al., 2019), insulin (Zhu et al., 2020), calcitonin (Tas et al., 2012) and influenza vaccine (Stinson et al., 2021). As portable and minimally invasive devices, MN patches that contain hundreds of MNs connecting with supporting layers can effectively overcome the barrier of stratum corneum to facilitate transdermal delivery of nanoparticles that are far greater than pure drugs, either by producing reversible microchannels for enhancing skin permeation of topically applied nanoparticles (e.g., solid MNs), or by getting dissolved under the skin to achieve direct delivery of nanoparticles in the skin (e.g., coated MNs and dissolvable MNs) (Figure 1). Although the topic about transdermal delivery of nanoparticles *via* MNs has been recently reported by some review papers (Chen et al., 2020; Alimardani et al., 2021; Ruan and Zhang, 2021; Salwa et al., 2021), this work put a different emphasis on this subject that include the design and summarization of drug-carrying nanoparticles integrated with MNs. In this review, we first introduce the four types of nanoparticles capable of carrying drugs, including nanocrystals, lipid nanoparticles, polymeric nanoparticles and inorganic nanoparticles, and then describe MN-based strategies that have been adopted to aid transdermal delivery of these nanoparticles for drug delivery. Finally, we provide our perspectives on the potential translation of MNs-mediated delivery of nanoparticles in the skin.

**TABLE 1:** The comparison of different administration routes for drug-carrying nanoparticles.

Administration routes	Microneedles	Oral	Intravenous	Topical
Advantages	No pain; self-administration; enabling localized drug delivery; High bioavailability; increase patient compliance; reduced side effects; low cost	Easy to use; no pain	High bioavailability	Easy to use; no pain
Drawbacks	Limited drug dose	Low bioavailability; poor distribution; requiring frequent administration; undesirable side effects	Pain; reduced patient compliance; requiring healthcare providers; systemic toxicity	Poor bioavailability; extremely low absorption; only for small lipophilic drugs use

## Nanoparticles for Drug Delivery Mediated by Microneedles

There have been a great number of drug-carrying nanoparticles that are developed for transdermal drug delivery mediated by MNs, either for localized delivery or for systemic release to treat a variety of diseases, such as skin cancer, contraception, diabetes or cardiovascular diseases (Chen et al., 2020; Salwa et al., 2021; Vora et al., 2021) (Table 2). These nanoparticles can be classified into four types based on their fabricating materials, including nanocrystals that are made of pure drugs, lipid-based nanoparticles that are fabricated with lipids, polymeric nanoparticles that are comprised of natural or synthetic polymers, and inorganic nanoparticles that are composed of inorganic materials (e.g., silicas, metals).

**TABLE 2:** The representative applications of transdermal delivery of drug-loaded nanoparticles *via* MNs.

Type of nanoparticle	Type of MNs	Carried drug	Application	References
Nanocrystals	Dissolvable MNs	Rilpivirine	Anti- human immunodeficiency virus (HIV)	Mc Crudden et al. (2018)
	Dissolvable MNs	Methotrexate	Treatment of psoriasis	Tekko et al. (2020)
Lipid-based nanoparticles	Dissolvable MNs	doxycycline, diethylcarbamazine and albendazole	Antifilariasis drugs	Permana et al. (2019)
	Coated MNs	Cisplatin	Anticancer	Lan, (2018)
Polymeric nanoparticles	Solid MNs	Insulin	Diabetes treatment	Zhang et al. (2020)
	Hollow MNs	Ovalbumin	Vaccine antigen	de Groot et al. (2017)
Inorganic nanoparticles	Dissolvable MNs	Doxorubicin	Anticancer	Dong et al. (2018)
	Coated MNs	Ovalbumin	Vaccine antigen	Tu et al. (2017)

### Nanocrystals

Generally, low bioavailability and small absorption of poorly soluble drugs represent major problems in the pharmaceutical drug development (Savjani et al., 2012). Numerous efforts have been made to increase the solubility and biodistribution of poorly soluble drugs (Nagarwal et al., 2011), among which nanocrystal technology plays an important role in addressing the problems associated with low solubility of drugs. Nanocrystals are carrier-free drug particles within nanometer size range, and have increased dissolution rate due to increased surface area, thus possessing enhanced bioavailability (Srivalli and Mishra, 2015). Moreover, owing to the structure of non-polymer covering, nanocrystals have high drug loading capability (as high as 100%), which makes them extremely attractive for treatments of the diseases that usually require high drug doses. A further characteristic of nanocrystals is that they can achieve sustained release of the drug for an extended period at the administration site due to the slow dissolution in the aqueous environment (Permana et al., 2020).

### Lipid-Based Nanoparticles

Lipid-based nanoparticles are most typically spherical vesicles with single or multi-lipid bilayers that encapsulate aqueous droplets, and the lipid-based nanoparticles mainly include: liposomes, nanoemulsions, solid lipid nanoparticles (SLNs), nanostructured lipid carriers (NLCs) (Vitorino et al., 2014). Due to the lipophicity of the nanoparticles, they are beneficial to increase solubility of poorly water-soluble drugs, thereby enhancing bioavailability. Besides, lipid layers of nanoparticles can fuse with stratum corneum lipids to further improve the drug transport through the skin (Kuntsche et al., 2008). As one kind of the most prevalent lipid-based nanocarriers, liposomes are usually made of phospholipids that can form unilamellar and multilamellar vesicular structures, which makes liposomes suitable to carry and deliver hydrophilic, hydrophobic or lipophilic drugs (Mitchell et al., 2021).

### Polymeric Nanoparticles

Polymeric nanoparticles used for drug delivery offer many special advantages over other kinds of nanoparticles, including increased target ability after surface modification, improved biocompatibility, reduced cytotoxicity and prolonged drug release duration (Begines and Ortiz, 2020). A variety of natural or synthetic polymers have been used in polymeric nanoparticle formulations, such as poly (D, l-lactide-co-glycolide) (PLGA), poly (lactic acid) (PLA), polyethylene glycol (PEG), polyacrylates, chitosan, alginate, gelatin and albumin (De Jong and Borm, 2008). Among the above polymers, PLGA is the most frequently used hydrophobic polymer because of its excellent biocompatibility and slow biodegradation rate, which makes it appealing for the development of controlled release formulations (Naves et al., 2017), and PEG is the most commonly used hydrophilic polymer with non-immunogenic, biocompatible and flexible nature, which makes it suitable for the delivery of hydrophilic drugs or bioactive molecules that usually require mild condition during encapsulation process (Jeyhani et al., 2019).

### Inorganic Nanoparticles

Inorganic nanoparticles are non-toxic, hydrophilic, biocompatible and highly stable, which makes them ideal for drug delivery. Inorganic nanoparticles for drug delivery mainly include mesoporous silica nanoparticles (MSNs), superparamagnetic iron oxides (SPIONs), quantum dots and metallic nanoparticles. MSNs have been widely used as controlled release carriers for drugs due to their large specific surface area, regular pore structure, adjustable pore size and good biocompatibility. Meanwhile, their unique mesoporous structure can prevent drugs from enzyme degradation or early release. In addition, the porous surface is covered with a large number of silica hydroxyl groups, which enables mesoporous silica nanoparticles to be functionalized by post-modification with a variety of polymers or specific drugs, forming intelligent drug control systems (Choi et al., 2021). SPIONs are kind of magnetic nanoparticles that can be guided by the direction of external magnetic field. Besides, they can also be used as contrast agents for magnetic resonance imaging (MRI) for diagnosis of diseases (Zhu et al., 2017). Quantum dots are semiconductor nanomaterials with diameters between 2 and 100 nm, usually prepared from III–V or group II–VI elements. Quantum dot excitation light has wide band range, narrow emission spectrum width, high fluorescence intensity, good stability, long life and certain antibacterial activity (Ren et al., 2020), which makes it have a good application prospect in wound healing (Salleh and Fauzi, 2021), drug transport (Fakhri et al., 2017), fluorescent biosensors (Hu et al., 2021) and disease diagnosis (Mansuriya and Altintas, 2021). In recent years, metallic nanoparticles have attracted growing interest in drug delivery, and the modification and functionalization of metallic nanoparticles with specific functional groups allow them to bind to antibodies, drugs and other ligands, making metallic nanoparticles promising in biomedical applications (Patra et al., 2018).

## Strategies of MN-Mediated Transdermal Delivery of Nanoparticles

Although many research have confirmed the benefits of nanoparticles as drug reservoirs for transdermal drug delivery, a lot of evidences demonstrate that nanoparticles still stay in the upper layer of stratum corneum and are restricted to deep penetration in the skin after topical application (Lademann et al., 2007; Sahle et al., 2017). In order to address the issue, minimally invasive MN-based strategies have been developed to facilitate transdermal delivery of therapeutics-loaded nanoparticles, including topical application of nanoparticles after MN penetration, transdermal delivery *via* coated MNs, transdermal delivery *via* dissolvable MNs, and transdermal delivery by the combination of iontophoresis and MNs.

### Topical Application Through the MN-Punctured Pores

**FIGURE 2:**
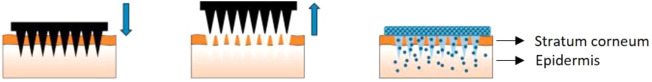
A schematic representation of solid MNs pretreatment for increasing the permeability of nanoparticles by creating micro-holes across the skin (Eneko et al., 2016).

The most typical strategy of MN-mediated transdermal delivery of nanoparticles is the topical application of a formulation containing nanoparticles after MNs pretreatment, which created microscopic puncture holes in the skin allowing nanoparticles to diffuse through the skin (Figure 2) (Eneko et al., 2016). Zhang et al. used confocal laser scanning microscopy to visualize the distribution of fluorescent PLGA nanoparticles in the skin through the microchannels produced by solid MNs application, and they observed a great number of nanoparticles could travel and deposit in *Epidermis* located below the stratum corneum, demonstrating the enhanced transdermal delivery of nanoparticles facilitated by solid MNs pretreatment (Zhang et al., 2010). This strategy has the advantage of enhancing skin permeation of nanoparticles, while it still suffers from limited drug amount that can be transported through the skin (Alimardani et al., 2021).

### Transdermal Delivery of Nanoparticles by Hollow MNs

Hollow MNs are also beneficial for transdermal delivery of nanoparticles, which allows for continuous delivery of liquid nanoparticle formulations, like nanoparticle suspensions, into the skin through the inserted hollow needles. Such kind of MNs is possibly capable of precisely delivering larger amounts of nanoparticles with spatial and temporal resolution compared to solid MNs (Roxhed et al., 2008). For example, Mir et al. developed a liquid injection system (AdminPen®) by combining bacterial enzyme-responsive nanoparticles with hollow MNs. *In vivo* skin insertion and dermatokinetic studies suggested that the system delivered about 8.5 times higher concentrations of the drug, carvacrol (CAR), in the form of NPs as compared with topically applied hydrogel containing pure CAR, indicating a great potential of increasing transdermal delivery of nanoparticles by hollow MNs (Mir et al., 2020). In spite of the capability of enabling transdermal delivery of precise and increased nanoparticles facilitated by hollow MNs, such strategy is compromised by the use of complicated setups.

### Transdermal Delivery of Nanoparticles *via* Coated MNs

**FIGURE 3:**
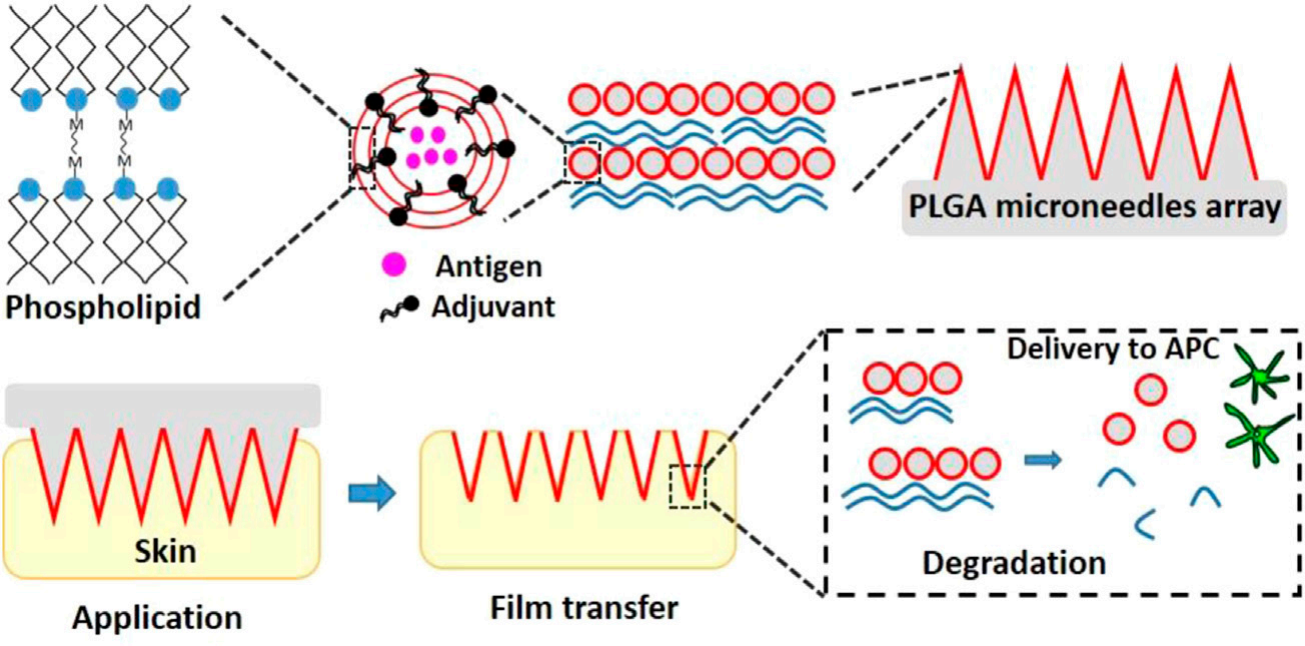
The schematic graph of PLGA MNs coated with PBAE and ICMVs for co-delivery of the antigen and adjuvant (DeMuth et al., 2012).

Coated MNs that contain a nanoparticle formulation at the surface can completely dissolve the coating and subsequently deliver the nanoparticles at the administration site upon skin insertion. Coated MNs are generally made of solid MNs and surface coatings that can be prepared by various methods such as dip coating (Ma and Gill, 2014), spray coating (Ning et al., 2020) and other sophisticated methods (Tort and Mutlu Agardan, 2020). For example, DeMuth et al. designed PLGA MNs coated with cationic poly (β-amino ester) (PBAE) and negatively charged interbilayer-cross-linked multilamellar lipid vesicles (ICMVs) for delivery of protein antigen and the antigen adjuvant (DeMuth et al., 2012). This coating of PBAE and ICMV rapidly transferred from the MNs to the skin after MNs insertion, leading to an efficient delivery of antigens to antigen-presenting cells and inducing a robust immune response (Figure 3) (DeMuth et al., 2012). Although this method is very straightforward, but it still suffers from limited amount of nanoparticles that can be delivered into the skin *via* coated MNs (Kim et al., 2012).

### Transdermal Delivery of Nanoparticles *via* Dissolvable MNs

**FIGURE 4:**
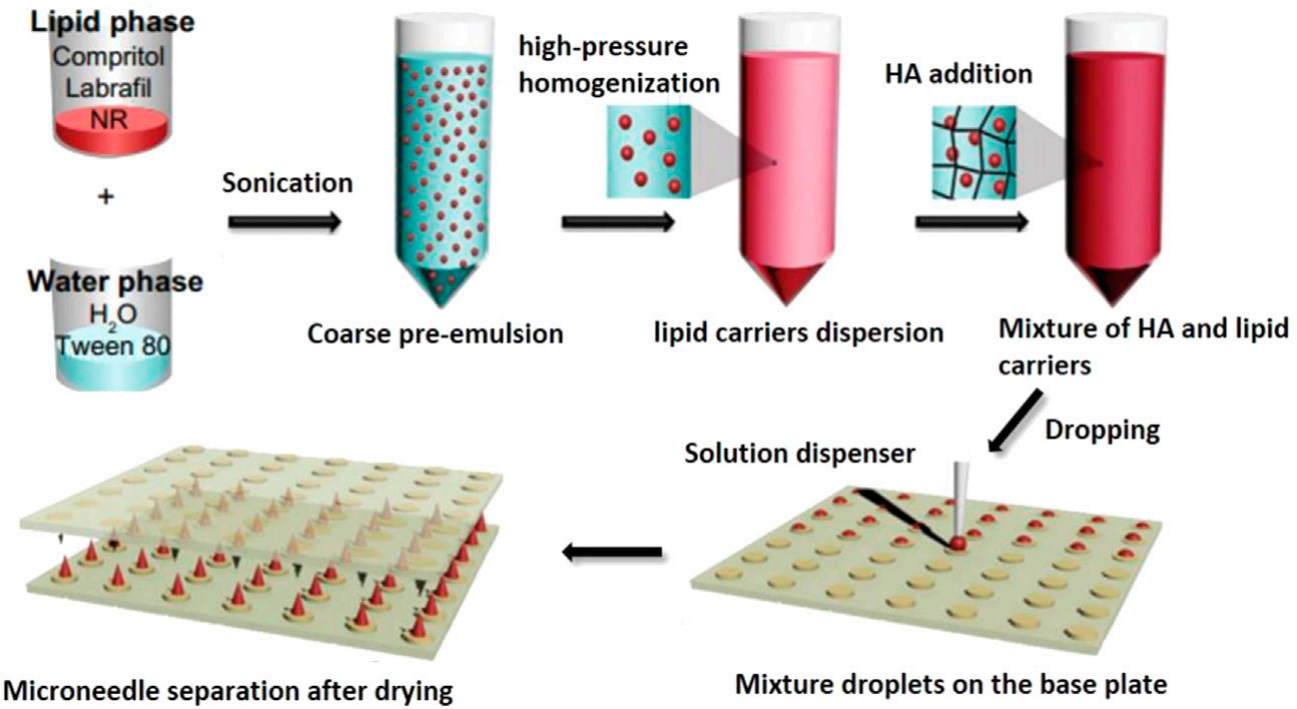
The schematic graph of the synthesis of HA MNs containing lipophilic NR-loaded lipid nanoparticles. Abbreviation: HA (hyaluronic acid), NR (Nile red), NLCs (nanostructured lipid carrier) (Lee et al., 2014).

The grim situation of poorly soluble drugs in the topical application has encouraged the combination of drug nanocrystals or drug-loaded nanoparticles with dissolvable MNs (Figure 4) (Lee et al., 2014). Unlike coated MNs, dissolvable MNs can get dissolution of the whole MNs and then release the encapsulated nanocrystals or nanoparticles at the administration site under skin, which makes dissolvable MNs be able to deliver more nanoparticles compared with coated MNs. For example, methotrexate sodium salt (MTX Na), a drug for psoriasis treatment, is poorly water soluble and hard to be used topically. Such drug could be made into nanocrystals and incorporated into dissolvable MNs. After skin insertion, the MTX nanocrystal-carrying MNs exhibited desired drug delivery efficiency, showing approximately 322-fold higher accumulation in the skin 24 h after administration than free MTX. *In vivo* studies in rats revealed that 72 h after administration, there was still about 12.5% of the MTX nanocrystals deposited in the skin, suggesting a localized and sustained drug delivery facilitated by the strategy of dissolvable MNs and nanotechnology (Tekko et al., 2020).

### Combination of Iontophoresis With MNs for Transdermal Delivery of Nanoparticles

Electric-field related methods have been used as auxiliary means for better drug transport (Niamlang and Sirivat, 2009). Transdermal iontophoresis is a physically noninvasive method that involves applying a low electrical potential gradient across the skin to facilitate the passage of charged or polar substances through the skin (Katikaneni et al., 2009). However, the use of iontophoresis alone still has limited improvement and do not significantly promote drug penetration from stratum corneum to deeper layers (e.g., Epidermis and dermis), especially for those biological macromolecules such as proteins or DNA. The strategy of combining iontophoresis with MNs can significantly improve the transdermal delivery efficiency as well as broaden the diversity of drugs ranging from small chemicals to large molecules or drug-loaded nanoparticles (Katikaneni et al., 2009; Lanke et al., 2009; Vemulapalli et al., 2012; Gaware et al., 2019). For example, Chen et al. investigated the transdermal delivery of insulin-loaded nanovesicles driven by iontophoresis through microchannels created by solid MNs (Chen et al., 2009). Facilitated by MNs puncture, *in vivo* permeation study exhibited 86.1–166.7 times higher drug permeability than that without MNs pretreatment. Further, under the influence of a forward current, the positive charged nanovesicles accelerated the movement towards deeper site of skin through the microchannels created by MNs and showed 3.4–7.1 times higher than nanovesicles with MNs pretreatment alone, suggesting the greatly improved delivery efficiency of nanoparticles when using MNs and iontophoresis together. Although this method possesses significantly enhanced efficiency for transdermal delivery of drug-carrying nanoparticles, it is only restricted to polar substance and has limited effect for neutral nanoparticles compared with the approach of MNs pretreatment alone.

## Conclusion and Perspectives

Due to the unique advantages, drug-carrying nanoparticles have been demonstrated to be valuable drug delivery systems in a variety of biomedical applications, and some drug-loaded nanoparticles have even been applied for clinical use (Mitchell et al., 2021). Although nanoparticles can be administered *via* different routes, like oral taken and intravenous injection, MN-mediated transdermal delivery of nanoparticles has attracted considerable interest since this administration method can significantly improve drug bioavailability while avoiding pain associated with hypodermic injection. In this review, we introduced four types of currently used nanoparticles for drug delivery and summarized the strategies that had been explored to facilitate the transdermal delivery of drug-loaded nanoparticles.

The development of microfabrication technology and nanotechnology will enhance drug stability during preparation of nanoparticles and fabrication of MNs, increase drug amount for each MN patch, and promote transdermal drug delivery efficiency after skin insertion. Also, special designs (e.g., core-shell structure) can be incorporated in the nanoparticle-encapsulated MNs, and facilitate the delivery systems to achieve sustained release of drugs for an extended period under the skin (e.g., 3 or 6 months), which will make the systems appealing for the treatment of chronic diseases by reducing dosing frequency and increasing patient compliance, such as type 2 diabetes, cancer, obesity, psoriasis or spinal cord injury. It is optimistically envisioned that expanded academic research in MNs and nanoparticles will accelerate clinical translation of MN-mediated delivery of nanoparticles for transdermal drug delivery.
